# Transient Receptor Potential Channels as an Emerging Target for the Treatment of Parkinson’s Disease: An Insight Into Role of Pharmacological Interventions

**DOI:** 10.3389/fcell.2020.584513

**Published:** 2020-11-20

**Authors:** Bhupesh Vaidya, Shyam Sunder Sharma

**Affiliations:** Department of Pharmacology and Toxicology, National Institute of Pharmaceutical Education and Research (NIPER), Mohali, India

**Keywords:** Parkinson’s disease, TRP channels, TRPM2, TRPV1, TRPC1

## Abstract

Parkinson’s disease (PD) is a neurodegenerative disorder characterized by the symptoms of motor deficits and cognitive decline. There are a number of therapeutics available for the treatment of PD, but most of them suffer from serious side effects such as bradykinesia, dyskinesia and on-off effect. Therefore, despite the availability of these pharmacological agents, PD patients continue to have an inferior quality of life. This has warranted a need to look for alternate strategies and molecular targets. Recent evidence suggests the Transient Receptor Potential (TRP) channels could be a potential target for the management of motor and non-motor symptoms of PD. Though still in the preclinical stages, agents targeting these channels have shown immense potential in the attenuation of behavioral deficits and signaling pathways. In addition, these channels are known to be involved in the regulation of ionic homeostasis, which is disrupted in PD. Moreover, activation or inhibition of many of the TRP channels by calcium and oxidative stress has also raised the possibility of their paramount involvement in affecting the other molecular mechanisms associated with PD pathology. However, due to the paucity of information available and lack of specificity, none of these agents have gone into clinical trials for PD treatment. Considering their interaction with oxidative stress, apoptosis and excitotoxicity, TRP channels could be considered as a potential future target for the treatment of PD.

## Introduction

Parkinson’s disease (PD) is a neurodegenerative condition characterized mainly by the loss of dopaminergic neurons in regions of the basal ganglia. It is the second most prevalent form of neurodegenerative disorder and stands next to Alzheimer’s disease (AD) in terms of number of individuals affected across the globe (Parkinson’s News Today, 2020). There are a number of therapeutics (Levodopa, Dopamine (D2) receptor agonists, monoamine oxidase B inhibitors, catechol-*o*-methyl transferase inhibitors, Adenosine 2A antagonists) approved for the treatment of PD. However, most of them provide only symptomatic relief. Also, currently available drugs show severe side effects and require frequent dosing resulting in low patient compliance of these medications. This has warranted a search for new mechanisms as well as molecular targets which could be utilized for the development of drug candidates in PD (Kumar et al., 2009; Uppalapati et al., 2014; Das and Sharma, 2015; Zhen and Chu, 2020). There are a number of *in vitro* and *in vivo* models which have been used for the therapeutic screening of pharmacological agents targeting these new molecular targets in PD. PD models include administration of neurotoxins such as 1-methyl-4-phenyl-1,2,3,6-tetrahy- dropyridine (MPTP), 1-methyl-4-phenylpyridinium (MPP^+^), rotenone, 6-hydroxydopmaine (6-OHDA) and alpha-synuclein pro-fibrils to induce dopaminergic neuronal death in the substantia nigra and the striatum. In addition, transgenic mice (knock-in and knock-out) are available for induction of PD. Pathophysiology of PD is complex and multi-factorial. Studies done using these preclinical models as well as PD patients have pointed out at the involvement of Ca^2+^ overload and oxidative stress in PD pathology. Cumulative evidence in recent times has demonstrated a major contribution of Transient Receptor Potential (TRP) channels toward Ca^2+^ mediated excitotoxicity and oxidative stress-induced dopaminergic neuronal death in PD. In total, 28 members of the TRP family have been discovered in mammals which are activated in response to diverse physiological stimuli (Zheng, 2013; Samanta et al., 2018). However, most of these members are non-selective cation channels which are responsible for the influx of monovalent and divalent ions, including Ca^2+^ into the cell. Oxidative stress and reactive oxygen species (ROS) have also been postulated to play a role in mechanisms involving activation and inhibition of these TRP channels (Adhya and Sharma, 2019; Akyuva and Naziroglu, 2020). These properties make them an attractive target for the treatment of neurodegenerative conditions, including PD (Thapak et al., 2020c). As a result, last few decades have seen enormous progress in the field of TRP research, and many of the TRP channel modulators like AMG571, MK-2295 and AZD1386 (TRPV1 antagonists) have even managed to progress to the clinical trials for the treatment of pain disorders (Kaneko and Szallasi, 2014). However, further analysis of these TRP channel modulators has exposed their dark side with side effects raging form hyperthermia and to reduced noxious perception to heat (Gavva, 2009; Kaneko and Szallasi, 2014). Thus, most of these studies had to be terminated owing to the risk of scalding injuries to the patients. Keeping in mind the crucial role of these channels, it is now expected that detailed structure-activity relationships and pharmacophore studies would help to improve their safety and efficacy and ensure the translational success of TRP channel modulators in PD.

In this review, we have provided a detailed account of preclinical evidence suggesting the vital involvement of TRP channels in PD and how their knowledge may help researchers in this field gain in-depth insight about these channels. Additionally, a number of upcoming drug candidates targeting TRP channels have also been discussed.

## TRP Family

TRP family of ion channels was first discovered in 1969 when some of the drosophila fly mutants turned blind in response to bright light (Cosens and Manning, 1969). This identification was followed by a series of biochemical, electrophysiological and molecular studies which led to the characterization of the TRP channel family. It is now well established that these channels are evolutionarily conserved across species and are found in most cells, tissues, and organisms. Subsequently, a total of 28 members belonging to 6 subfamilies were identified in mammals which include TRPV (Vanilloid), TRPM (Melastatin), TRPC (Canonical), TRPML (Mucolipin), TRPA (Ankyrin), and TRPP (Polycystic) (Li, 2017). Besides, other families, such as TRPY and TRPN were also identified in the non-mammalian species. Amongst these, TRPY is the most distant relative which is only present in yeast. It plays a role in the release of Ca^2+^ from the vacuoles when exposed to hyperosmotic conditions (Denis and Cyert, 2002; Venkatachalam and Montell, 2007). TRPN has also been reported but only in a limited number of vertebrates such as xenopus and zebrafish, where it serves as the mechanosensitive channel for performing the normal physiological function (Hardie, 2007). However, these channels bear little relevance to human disease and hence have not been discussed in greater detail. Structurally all the TRP channels form six transmembrane helixes with both the N and C terminal located within the cytoplasm. These channels are activated in response to a diverse range of physiological and sensory stimuli which include mechanical, thermal, electrical and chemical cues (Steinberg et al., 2014). Therefore, these TRP channels are involved in the sensation of touch, vision, olfaction, temperature and nociception (Voets et al., 2005). Moreover, these TRPs exhibit a widespread expression in the brain areas involved in pathophysiology of PD, making them a very relevant molecular target for the discovery of new drugs to treat PD (Table 1). The detailed role of TRP channels has been discussed in the further sections.

**TABLE 1:** Expression of the different TRP channels in the regions involved in the pathophysiology of PD.

S No.	TRP channel	Substantia nigra pars compacta	Striatum	Subthalamic nuclei	Substantia nigra pars reticulata	References
1	TRPV1^c^	+	+	+	nd	Roberts et al., 2004; Musella et al., 2009; Nam et al., 2015
2	TRPV2*^nk^	nd	+	nd	nd	Kunert-Keil et al., 2006; Shibasaki et al., 2013
3	TRPV3^nk^	+	+	nd	nd	Xu et al., 2002; Guatteo et al., 2005
4	TRPV4^rd^	+	+	nd	nd	Guatteo et al., 2005; Tjaša et al., 2016
5	TRPV5*^nk^	nd	nd	nd	+	Kumar et al., 2017
6	TRPV6^nk^	+	+	nd	+	Lehen’kyi et al., 2012
7	TRPM1^nk^	nd	nd	nd	nd	Fonfria et al., 2006
8	TRPM2^id^	+	+	+	+	Fonfria et al., 2005; Lee et al., 2013; Yu et al., 2014
9	TRPM3*^nk^	nd	nd	nd	nd	Lee et al., 2003
10	TRPM4^nk^	+	+	nd	nd	Mrejeru et al., 2011; Chen et al., 2019
11	TRPM5*^nk^	nd	nd	nd	nd	Kunert-Keil et al., 2006
12	TRPM6*^nk^	nd	nd	nd	nd	Kunert-Keil et al., 2006
13	TRPM7^id^	+	+	nd	nd	Fonfria et al., 2005; Yu et al., 2014
14	TRPM8^nk^	+	nd	nd	nd	Ordás et al., 2019
15	TRPC1^rd^	+	+	+	+	Martorana et al., 2006; Sun et al., 2017
16	TRPC2^nk^	nd	nd	nd	nd	
17	TRPC3^rd^	+	+	+	+	Chung et al., 2007
18	TRPC4^nc^	+	+	+	+	Chung et al., 2007
19	TRPC5^nc^	+	+	+	+	Chung et al., 2007
20	TRPC6^nc^	+	+	+	+	Chung et al., 2007
21	TRPC7^nk^	+	+	+	+	Chung et al., 2007
22	TRPML1*^nk^	nd	nd	nd	nd	Anglade et al., 1997; Dong et al., 2008; Grimm et al., 2012
23	TRPML2^nk^	nd	nd	nd	nd	
24	TRPML3^nk^	nd	nd	nd	nd	
25	TRPA1^nk^	nd	+	nd	nd	Bodkin et al., 2014
26	TRPP1^nk^	nd	nd	nd	nd	
27	TRPP2^nk^	nd	nd	nd	nd	
28	TRPP3^nk^	nd	nd	nd	nd	

^+^Expression is this region has been determined. ^*nd*^Expression in this region has not yet been determined. *Expression in basal ganglia has been characterized but individual distribution within basal ganglia has not been reported in much detail. ^*c*^There is a controversy related to whether the expression is increased or reduced in PD. ^*nk*^Change of expression in PD has not yet been studied. ^*id*^Expression is known to be increased in PD. ^*rd*^Expression is known to be reduced in PD. ^*nc*^No change of expression in PD.

## Contribution of TRP Channels to PD

### TRPV Involvement in PD

TRPV channels are abundantly expressed in several areas of the brain. Although different members of the TRPV subfamily perform a very diverse physiological role, all of these have modest permeability toward Ca^2+^. It makes them important for various central nervous system (CNS) functions, including learning and memory (Thapak et al., 2020a). It has therefore been reported that any change in the expression of TRPV channels contributes toward CNS disease pathologies such as AD, PD, cerebral ischemia, depression and anxiety (Bashiri et al., 2018; Thapak et al., 2020c).

**FIGURE 1:**
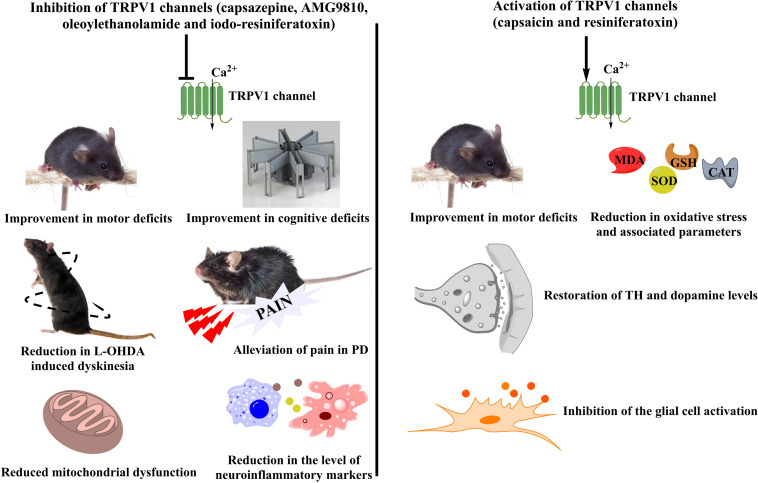
Controversial role of TRPV1 channels in PD. A number of TRPV1 modulators have been tested for their beneficial effects in PD. Different agents working either via TRPV1 inhibition or TRPV1 activation have been studied for the mitigation of PD symptoms in preclinical models. Inhibition of TRPV1 led to an improvement in the motor and cognitive deficits of PD accompanied by reduced mitochondrial dysfunction and decreased levels of inflammatory mediators. Moreover, TRPV1 inhibitors also showed a reduction in the 6-OHDA induced dyskinesias and provided relief from pain which is one of the major non-motor symptoms of PD. On the other hand, TRPV1 activation had similar effects on improvement of motor coordination. It also reduced the levels of oxidative stress and led to the restoration of the tyrosine hydroxylase and dopamine levels. Furthermore, a significant reduction in the microglial activation was also observed after the administration of TRPV1 activators. TRPV1, Transient Receptor Potential Vanilloid 1; PD, Parkinson’s disease; 6-OHDA, 6-hydroxydopamine; TH, Tyrosine hydroxylase; MDA, Malondialdehyde; SOD, superoxide dismutase; GSH, glutathione; CAT, catalase.

Amongst the members of the TRPV family, TRPV1/4/5 have been studied for their involvement in PD pathology. TRPV1 is expressed in the regions, striatum and substantia nigra, which are known to be affected in PD (Musella et al., 2009; Nam et al., 2015). However, its role remains controversial pertaining to modulation of different downstream signaling pathways by it. Though an upregulation of TRPV1 has been reported by several authors in different PD models, both agonists and antagonists targeting these have been successful in alleviating symptoms of disease in the preclinical stages (Figure 1) (Nam et al., 2015; Li et al., 2019).

Studies related to other TRPV channels like TRPV4/5 have also been carried out but remain rather limited. Expression of TRPV4 was downregulated in SNpc and striatum of rats after 6-OHDA administration suggesting a possible correlation between TRPV4 expression and neuronal death (Tjaša et al., 2016). Similarly, there is not much data to support the involvement of TRPV5 channels in PD. However, a transcriptome analysis suggested its possible role in the regulation of the pathophysiological hallmarks of PD in 6-OHDA treated rats (Li et al., 2019). Nevertheless, the lack of enough data and the absence of any pharmacological study targeting these channels have denied them a concrete claim as a possible drug target for PD.

### TRPM Involvement in PD

TRPM channel subfamily has been extensively explored for its potential involvement in pathophysiology of several neurodegenerative disorders including PD (Ostapchenko et al., 2015; Sun et al., 2018b; Thapak et al., 2020b). It has been reported that the members of the TRPM subfamily lead to an influx of monovalent and divalent cations inside the cell which might serve as a contributing factor for the excitotoxicity mediated neuronal death (Adhya and Sharma, 2019).

Amongst these, TRPM2 channels have gained importance for their involvement in several mechanisms related to neuronal death. These channels are activated in response to oxidative stress and adenosine diphosphate ribose (ADPR), both of which are increased in PD. Though the permeability ratio of TRPM2 for Ca^2+^ to Na^+^ (PCa:PNa) is nearly 0.7 indicating a higher permeability for Na^+^, a substantial Ca^2+^ influx also takes place because of the longer opening time of TRPM2 channels (Belrose and Jackson, 2018). The increased Ca^2+^ levels as a result of TRPM2 activation lead to excitotoxicity and contribute toward the aggravation of the underlying PD pathology.

Apart from TRPM2, TRPM7 has also been investigated for its involvement in PD. Genetic variants of TRPM7 have been linked to the ionic dyshomeostasis, mitochondrial dysfunction, inflammation and increased oxidative stress, all of which led to its identification as a candidate susceptibility gene for familial PD (Hermosura et al., 2005; Hermosura and Garruto, 2007). In addition, PC-12 cells exposed to 6-OHDA also showed an increased expression of TRPM7, which was reduced after overexpression of miR-22. Furthermore, miR-22 overexpression also protected from the effects of TRPM7 upregulation by inhibition of apoptosis, reduction of ROS and improved cell viability (Yang et al., 2016). Despite these findings, there are other controversial reports which have observed neuroprotective effects of the TRPM7 mediated Mg^2+^ influx in the MPP^+^ based *in vitro* model (Shindo et al., 2015). Hence, more studies with selective TRPM7 modulators are required to conclusively elucidate the role of these channels in PD.

TRPM8 is another candidate belonging to the TRPM subfamily which has been identified as a risk factor for pain in PD (Williams et al., 2020). Pain is a frequently occurring non-motor symptom, which has been reported in about 86% of the PD patients (Beiske et al., 2009; Skogar and Lokk, 2016). Therefore, strategies targeting TRPM8 channels could be utilized in future for the management of pain in PD patients.

**FIGURE 2:**
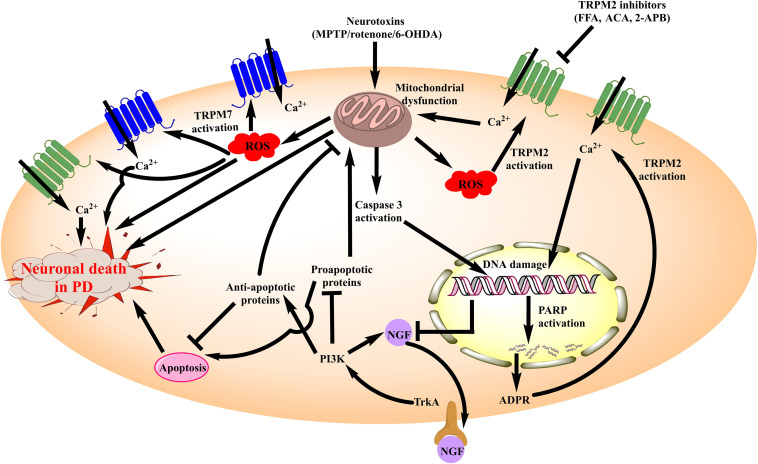
Schematic diagram illustrating the role of TRPM channels in neuronal death associated with PD. Amongst the TRPM channels, TRPM2 and TRPM7 have been elucidated for their involvement in PD. Neurotoxins such as MPTP, 6-OHDA and rotenone inhibit the mitochondrial complexes producing mitochondrial dysfunction. It generates ROS which acts as an activator of both TRPM2 and TRPM7 leading to the increased Ca^2+^ influx within neurons. These events in totality increase the neuronal death in PD besides aggravating mitochondrial dysfunction. Moreover, cytochrome c (Cyt c) is released from the mitochondria in this process which promotes pro-apoptotic signaling and damages DNA. Damage to the DNA leads to poly ADP ribose polymerase (PARP) mediated ADPR formation which is also an activator of TRPM2 channel. Therefore, the cascade of events exhibits a positive feedback loop which aggravates neuronal death. Besides, there is a reduced expression of NGF and associated TrkA/PI3K signaling, which intensifies the pro-apoptotic response and plays its part in the TRPM2 and TRPM7 mediated neuronal death in PD. MPTP, 1-methyl-4-phenyl-1,2,3,6-tetrahydropyridine; 6-OHDA, 6-hydroxy dopamine; ROS, reactive oxygen species; FFA, Flufenamic acid; ACA, *N*-(*p*-amylcinnamoyl) anthranilic acid; 2-APB, 2-Aminoethoxydiphenyl borate; TRPM, Transient receptor potential melastatin 2; NGF, Nerve growth factor; PARP, poly ADP ribose polymerase; PI3K, phosphoinositide 3-kinase; ADPR, Adenosine di-phosphate ribose; TrkA, Tropomyosin receptor kinase A.

In totality, these findings suggest that TRPM channels are critical molecular players for neuronal death in PD (Figure 2). However, the dearth of information related to their signaling pathways has largely limited the development of selective modulators for these channels in the PD models.

### TRPC Involvement in PD

TRPC subfamily has a widespread distribution in the CNS. It was also shown to exhibit high expression in the areas of SNpc and striatum, both of which are involved in the pathophysiology of PD (Sukumaran et al., 2017). As a result, different members of the TRPC subfamily have been investigated for their potential involvement in PD. TRPC1 was the first member of the TRPC subfamily to be identified in mammals. It is localized to the dendritic processes and co-expressed with tyrosine hydroxylase (TH) in the dopaminergic neurons of the SNpc (Martorana et al., 2006). This pointed out at the physiological relevance of TRPC1 channels in the dopaminergic system and raised the possibility that these channels may contribute toward the pathogenesis of PD. Studies carried out using the MPP^+^/MPTP model showed that the expression of TRPC1 was reduced in both the SNpc as well as PC12 neuronal cells in PD. Besides, TRPC1^–/–^ mice also reported motor deficits and increased Terminal deoxynucleotidyl transferase dUTP nick end labeling (TUNEL)-positive cells in the basal ganglia suggesting the critical role of TRPC1 in regulating apoptotic signaling within the cell (He et al., 2016). Similar results were observed in other studies where TRPC1 overexpression protected from the effects of 6-OHDA, MPP^+^ and α-synuclein toxicity in the neuronal cells (Bollimuntha et al., 2005; Sukumaran et al., 2018).

Several groups then looked at the downstream signaling associated with the neuroprotection offered by TRPC1 channels in PD models (Selvaraj et al., 2012). One of the possible mechanisms for this was the reduced endoplasmic reticulum (ER) stress which is otherwise increased in PD (Deng and Bai, 2012). It was shown that overexpression of TRPC1 channels in the SH-SY5Y cells treated with MPP^+^ led to the restoration of phosphatidylinositol-3-kinase (PI3K)/mammalian target of rapamycin (Akt/mTOR) signaling and increased dopaminergic neuronal survival. Interestingly, similar findings were reported in the brains of the PD patients and TRPC1^–/–^ mice which further showed an increased unfolded protein response (UPR) and reduced number of dopamine neurons in the brain (Selvaraj et al., 2012). On the other hand, TRPC1 deletion mediated ER stress was found to be associated with increased ROS, dysregulation of the glucose-regulated protein 78 (GRP78) and alteration in protein kinase RNA-like ER kinase (PERK) signaling pathways (Wang et al., 2018). In addition, potential crosstalk between the TRPC1-Stromal interaction molecule (STIM) and L-type calcium channels was also established using an electrophysiological study in the SNpc of the TRPC1^–/–^ mice (Sun et al., 2017). As L-type calcium channel blockers like isradipine have already gone into clinical trials for PD, TRPC1 performing the same physiological function in dopaminergic neurons could be looked at as a molecular target in PD (Simuni et al., 2010; Sun et al., 2017). Consistent with these results are the findings of Sun et al. (2018a), where MPP^+^ was found to reduce TRPC1 expression and store-operated calcium entry in the mesenchymal stem cell-derived dopaminergic neurons.

**FIGURE 3:**
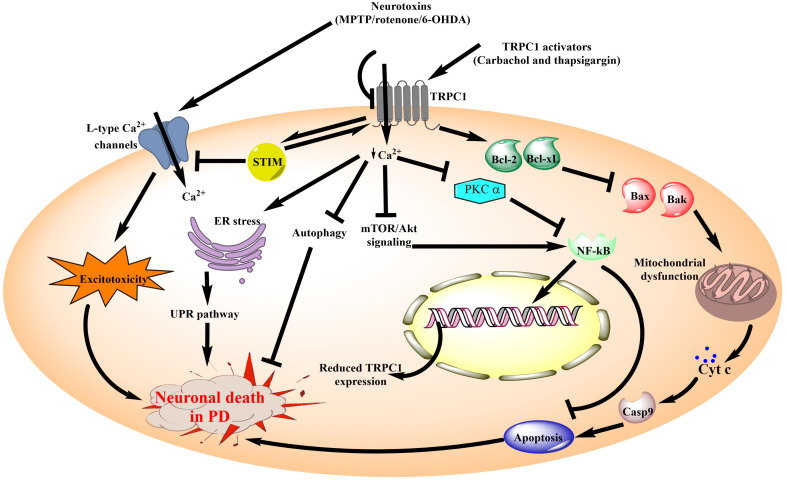
Schematic diagram illustrating the role of TRPC1 channels in neuronal death associated with PD. TRPC1 channel is the most widely studied member of the TRPC subfamily for its involvement in PD. Ca^2+^ influx through TRPC1 channels is known to be neuroprotective in the PD models. Additionally, review of literature suggests that inhibition of Ca^2+^ influx from TRPC1 by the administration of neurotoxins causes aggravation of the PD pathology. It leads to inhibition of mTOR/Akt signaling as well as reduced levels of PKC α. These events reduce NF-κB expression which in turn increases the DNA damage and pro-apoptotic signaling to compromise neuronal survival. On the other hand, TRPC1 activation directly promotes anti-apoptotic signaling to reduce mitochondrial dysfunction and associated cell death in PD. In addition, diminished Ca^2+^ influx from the TRPC1 channels results in impaired autophagy and ER stress. Moreover, in association with STIM, TRPC1 channels inhibit the Ca^2+^ influx via L-type calcium channels which are one of the major contributors toward excitotoxicity mediated neuronal death in PD. MPTP, 1-methyl-4-phenyl-1,2,3,6-tetrahydropyridine; 6-OHDA, 6-hydroxy dopamine; TRPC1, Transient Receptor Potential Canonical 1; ER, endoplasmic reticulum; STIM, Stromal interaction molecule; UPR, unfolded protein response; mTOR, mammalian target of rapamycin; Akt, Protein kinase B; PKC, Protein kinase C; NF-κB, nuclear factor kappa-light-chain-enhancer of activated B cells; Cyt c, cytochrome c; Casp9, caspase 9; Bcl2, B-cell lymphoma 2; Bcl-xl, B-cell lymphoma-extra large; Bax, Bcl2-associated X protein; Bak, Bcl-2 homologous antagonist/killer.

Other pathways that were affected in PD due to reduced TRPC1 expression include attenuation of autophagy as well as impairment of nuclear factor kappa-light-chain-enhancer of activated B cells (NF-kB) and Tropomyosin receptor kinase A (TrkA) signaling (Yu et al., 2013; Sukumaran et al., 2018). In contrast to these findings, downregulation of STIM, a TRPC1 activator was shown to prevent oxidative stress and apoptosis mediated neuronal death in 6-OHDA treated PC-12 cells (Li et al., 2014; Sukumaran et al., 2017). However, despite this discrepancy, most of the other studies have suggested a beneficial role of TRPC1 channels in the pathophysiology of PD are shown in Figure 3.

Other TRPC channels such as TRPC3 are also expressed in the SNpc and striatal regions of the brain. These are important for the regulation of firing intensity and neuronal depolarization in these brain regions (Zhou et al., 2008; Xie and Zhou, 2014). Additionally, these channels are also required for synaptic transmission and motor coordination, making them an important target for investigation in the context of PD (Hartmann et al., 2008). Initial observation though suggested little to no change in the TRPC3 expression in PD, studies carried out much later have suggested otherwise (Selvaraj et al., 2009, 2012; Yu et al., 2013; Streifel et al., 2014). In the 6-OHDA model, data from RT-PCR studies showed reduced expression of TRPC3 in the SNpc (Yu et al., 2013). Similar results were reported by other groups which revealed that the inhibitory effects of neurotoxins, MPP^+^ and 6-OHDA on TRPC3 was mediated through alteration in the purinergic calcium signaling (Lei et al., 2008; Streifel et al., 2014). However, drugs targeting TRPC3 have shown neuroprotective effects further challenging the role of molecular mechanisms related to TRPC3 in PD.

Besides the channels discussed in detail, most studies have invariably found no change in the expression of other TRPC channels including TRPC4/5/6 in different PD models as well as patients (Selvaraj et al., 2012; Lortet et al., 2013; Sukumaran et al., 2018). But, their ability to regulate the influx of calcium and other cations into the cell makes these channels an attractive candidate for studies in future.

### TRPML Involvement in PD

TRPML channels are non-selective cation channels localized intracellularly on the endosomes and lysosomes. These are responsible for the intracellular calcium influx after activation by different cellular pathways (Grimm et al., 2012). Amongst the members of TRPML subfamily, mutations in TRPML1 have also been linked to the lysosomal storage disorder, mucolipidosis type IV (MLIV) which is also a form of neurodegeneration. It is known to produce motor and cognitive deficits, similar to those seen in PD patients (Dong et al., 2008). Moreover, the role of TRPML1 in the autophagy-related signaling has also been elucidated, making it an important channel for cell survival. Although there is no direct evidence as of now for its involvement, it has been hypothesized that TRPML1 activation could restore the impaired autophagy seen in PD patients (Anglade et al., 1997). Furthermore, mutations in GBA gene, which codes for lysosomal enzyme glucocerebrosidase is one of the potential risk factors for PD and has also been known to cause lysosomal dysfunction (DePaolo et al., 2009). Therefore, there are several studies currently underway to investigate the possible role of TRPML channels in PD (The Michael J. Fox Foundation for Parkinson’s Research, 2019^1^; The Michael J. Fox Foundation for Parkinson’s Research, 2020^2^). These studies are expected to provide deeper insights into the pathophysiology of PD and help in the identification of new molecules which could be useful for PD management.

### TRPA Involvement in PD

TRPA channel subfamily remains relatively unexplored in the context of PD. Though there is no study pertaining to its direct involvement, agents acting on these channels have provided important clues related to the development of side effects associated with current PD therapy. These have been discussed ahead in much more detail.

## Potential Pharmacological Interventions Targeting TRP Channels in PD

A number of pharmacological agents have shown protective effects across different *in vitro* and *in vivo* studies. Detailed investigations pertaining to the beneficial effect of these agents has been discussed ahead and in Table 2.

**TABLE 2:** TRP channel modulators which accorded neuroprotection in the different PD models.

S No	TRP channel	Model	Pharmacological interventions	Major outcome	References
1	TRPM2	MPP^+^	Flufenamic acid and *N*-(*p*-amylcinnamoyl) anthranilic acid	• Inhibition of ROS production and apoptosis • Increased neuronal cell viability by TRPM2 inhibition *in vitro*	Sun et al., 2018b
2	TRPM2	Rotenone	Flufenamic acid and 2-Aminoethoxydiphenyl borate	Inhibition of oxidative stress	Nazıroğlu et al., 2011
3	TRPV1	MPTP and 6-OHDA	Capsaicin	• Reduction in microglial activation, inflammation and oxidative stress • Improvement in neurobehavioral parameters and restoration of TH levels in the dopaminergic neurons	Chung et al., 2017; Zhao et al., 2017
4	TRPV1	Drosophila fly mutants expressing human α-synuclein	Capsaicin	• Reduction in oxidative stress and restoration of dopamine levels • Improvement in climbing ability	Siddique et al., 2012, 2018
5	TRPV1	6-OHDA	AMG9810	• Reduced neuronal death • Improved performance in the rotarod test	Razavinasab et al., 2013
6	TRPV1	6-OHDA	Capsazepine and Oleoylethanolamide	• Reduction in L-DOPA induced dyskinesias through crosstalk with endocannabinoid signaling	Morgese et al., 2007; González-Aparicio and Moratalla, 2014
7	TRPV1	AAV-A53T knock in mouse	Resiniferatoxin	• Improvement in motor function • No change in the dopamine levels	Sorrento Therapeutics Inc, 2020
8	TRPC1	MPP^+^/MPTP	Carbachol and thapsigargin	• Accorded neuroprotection by increased AKT1 phosphorylation	Selvaraj et al., 2012
9	TRP3/6/7	MPP^+^	SKF-96365	• Reduction of intracellular Ca^2+^ overload by reducing the overexpression of post synaptic scaffolding protein Homer1	Chen et al., 2013

### TRPV1

A number of TRPV1 modulators have been studied by different groups in PD models. These include TRPV1 agonists (capsaicin and resiniferatoxin) as well as TRPV1 antagonists (capsazepine, AMG9810, oleoylethanolamide and iodo-resiniferatoxin). These agents exerted beneficial effects in the PD models. Capsaicin is an active phytochemical present in several *Capsicum* spp. which is highly selective toward TRPV1 channels (EC_50_ = 0.71 μM). It has been studied extensively for its disease-modifying effects in PD (Caterina et al., 1997; Musfiroh et al., 2013). It accorded neuroprotection in both the *in vitro* and *in vivo* rodent systems as well as in the drosophila model of PD. Capsaicin was further shown to improve rotarod performance and led to the restoration of dopaminergic signaling in the MPTP treated mice. Additionally, it also prevented glial cell activation and reduced oxidative stress in the astrocytes of MPTP induced PD mice. These effects were reversed after treatment with TRPV1 antagonists (capsazepine and iodo-resiniferatoxin) which confirmed that the beneficial effects were indeed attributed to the activation of TRPV1 channels (Chung et al., 2017). Similar observations were made in a study by Nam et al., which demonstrated that neuroprotective effects of capsaicin on TRPV1 were mediated through endogenous ciliary neurotrophic factor (CNTF) levels and CNTF-α receptors (Nam et al., 2015). It has been shown that the symptoms of PD first appear after 60–70% of the dopaminergic neuronal death has already taken place (Cheng et al., 2010). In a study, capsaicin was found to be capable of restoring the dopaminergic function even after the complete injury to the substantia nigra pars compacta (SNpc) and striatum (Kim et al., 2019). These results suggest the possibility for capsaicin to be effective in the later stages of PD when traditional medications fail to work. Besides, capsaicin also exerted a positive modulatory effect against oxidative insults and reversed the behavioral deficits induced by MPTP and 6-OHDA in the rodent models (Fong et al., 2016; Zhao et al., 2017). Capsaicin treated animals showed an increase in distance traveled in the open field and a significant reduction in the amphetamine-induced rotations after induction of PD. It was accompanied by a significant reduction in the malondialdehyde (MDA) levels and restoration of the superoxide dismutase (SOD) and catalase activity in the SNpc (Nam et al., 2015; Zhao et al., 2017).

The neuroprotective effects of capsaicin were further replicated in the drosophila fly mutants, which expressed a gene for human α-synuclein. These PD flies displayed a progressive form of neurodegeneration characterized by loss of dopamine function, oxidative stress and impaired performance in the climbing assay. Reversal of the pathological changes occurred in a dose-dependent manner which confirmed the therapeutic potential of capsaicin through TRPV1 activation for PD treatment (Siddique et al., 2012, 2018).

In contrast to these studies, other groups have shown that blockade of TRPV1 channels is also helpful in relieving some of the PD symptoms. A study involving the use of AMG9810, a selective TRPV1 blocker demonstrated a positive attenuation of the motor deficits after 6-OHDA administration which was evident from the rotarod test. Additionally, cresyl violet staining also showed that there was reduced neuronal death in the SNpc of the AMG9810 treated animals (Razavinasab et al., 2013). In line with the same, another TRPV1 blocker, capsazepine also exhibited neuroprotective effects in a number of PD models. Moreover, it was also found to reduce the occurrence of levodopa (L-DOPA) induced dyskinesia which is a prominent side effect seen with L-DOPA therapy in PD (Morgese et al., 2007). The most probable explanation for these effects came from a study where it was observed that TRPV1 blockade positively regulates the crosstalk between endocannabinoid receptors and TRPV1 downstream signaling which helps prevent L-DOPA induced dyskinesias (Lee et al., 2006; Giuffrida and Morgese, 2007; Morgese et al., 2007; Dos-Santos-Pereira et al., 2016). It is also supported by the findings in which oleoylethanolamide, another TRPV1 blocker showed protection in the 6-OHDA induced hemiparkinsonian model (González-Aparicio and Moratalla, 2014). Furthermore, blockade of TRPV1 has also been elucidated to play a crucial role in the modulation of pain pathways in PD (Li et al., 2020). TRPV1 expression was upregulated in the trigeminal subnucleus caudalis (Vc) which plays a role in central terminal sensitization of primary nociceptive neurons. In addition, its co-expression with 5-HT3A receptors in the rat dorsal root ganglion (DRG) and ability of TRPV1 blockers to inhibit the pain induced by 5-HT3A receptor agonists makes it a useful target for pain modulation in PD (Zeitz et al., 2002; Li et al., 2020).

Despite these promising results, the therapeutic window for the beneficial effects of TRPV1 modulators is very narrow and higher doses of TRPV1 blockers such as AMG9810 have been associated with learning and memory impairments in the preclinical studies (Razavinasab et al., 2013). Moreover, its interaction with endocannabinoids raised concern over the possible addictive potential and development of tolerance for these agents. However, when investigated, no such association was observed in studies involving TRPV1 blockers which makes them a viable option for studies in future (González-Aparicio and Moratalla, 2014).

In totality, these shreds of evidence suggested that targeting TRPV1 channels could be of therapeutic relevance for combating the motor and non-motor symptoms of PD. Therefore, to provide more proof to the speculation, Capsaicin is being investigated in patients for the elucidation of mechanisms related to swallowing and cough dysfunction in PD^3^. Another TRPV1 agonist, resiniferatoxin was shown to be effective in controlling the motor symptoms in the AAV-A53T knock-in PD mouse model (Sorrento Therapeutics Inc, 2020). According to the recent reports, it is soon expected to enter clinical trials which could open up new avenues of research for the management of PD.

Together, these studies capture a basic insight into the pharmacological modulation of TRPV channels which could be targeted in future for the treatment of PD. But, as the doubts over the clear molecular mechanisms, clinical efficacy and specificity persist, more detailed investigations are still necessary for the development of TRPV channel modulators as drug candidates in future.

### TRPM2

Studies conducted in preclinical models as well as PD patients, showed an increase in TRPM2 expression, suggesting its possible involvement in PD (Sun et al., 2018b; An et al., 2019). It was also demonstrated that MPP^+^ exposure leads to increased levels of TRPM2 accompanied by elevated oxidative stress and increased apoptosis in the SH-SY5Y neurons. It further resulted in reduced cell viability and caspase 3 activation in these neuronal cells (An et al., 2019; Ding et al., 2019). These effects were reversed by treatment with TRPM2 blockers, flufenamic acid (FFA) and *N*-(*p*-amylcinnamoyl) anthranilic acid (ACA). Moreover, similar results were obtained when knockdown of TRPM2 was carried out using a siRNA which resulted in reduced neuronal death and inhibition of apoptosis in the SH-SY5Y cells (Sun et al., 2018b). The detailed mechanism of this neuroprotection was delineated later in another study which attributed it to the working network of long non-coding RNA, p21 and microRNA, miR-625 present inside the neurons. It was also reported that long non-coding RNA, p21 is a positive modulator of TRPM2 expression and its knockdown could rescue from the toxicity induced by MPP^+^ treatment (Ding et al., 2019).

Other authors have also confirmed the involvement of TRPM2 in PD with the help of different toxin-based models *in vitro* and *in vivo*. In a study by Yu et al. (2014) it was shown that TRPM2 expression was significantly increased in the SNpc of 6-OHDA treated rats. This increased expression was attenuated by treatment with nerve growth factor, which accorded neuroprotection through phosphatidylinositol 3-kinase (PI3K) signaling pathway (Yu et al., 2014). Furthermore, it was demonstrated that TRPM2 mediated increased ROS production and caspase 3 activity was suppressed by increased expression of transcription factor GATA3 binding protein (GATA3) (Zhou and Han, 2017). This suggests the involvement of multiple downstream pathways affected by TRPM2 associated neuronal death in PD. In line with these shreds of evidence, rotenone also led to increased TRPM2 activation by ROS dependent mechanisms (Freestone, 2009). These effects were reversed after treatment with TRPM2 blockers FFA and 2-aminoethoxydiphenyl borate (2-APB), which confirmed the potential of TRPM2 blockade in the treatment of PD (Nazıroğlu et al., 2011). Furthermore, electrophysiological findings have revealed that increased spontaneous firing of the substantia nigra pars reticulata (SNr) GABAergic neurons seen in PD is modulated by increased activation of the TRPM2 channels (Lee et al., 2013). Conclusively, the close association of TRPM2 and intracellular ionic homeostasis has been established and any change in it makes the individual more susceptible toward PD (Hermosura and Garruto, 2007). Though there is ample evidence available for its involvement, there is no report which has investigated the effect of pharmacological interventions targeting TRPM2 *in vivo*. This has largely hindered the development of more potent analogs which could be tried for their translational potential of TRPM2 in PD. Further, the neuroprotective potential of TRPM2 antagonists in neurodegenerative disorders like AD strengthens the claim of TRPM2 antagonists as neuroprotective agents (Thapak et al., 2020b).

### TRPC1

Silencing of TRPC1 as well as its functional blockade was also associated with mitochondrial dysfunction and decrease in the TH levels after sub-chronic administration of MPTP to the C57BL/6 mice. These pathological changes were reversed after treatment with TRPC1 activators carbachol and thapsigargin or after TRPC1 overexpression which led to an increase in anti-apoptotic signaling (Selvaraj et al., 2009). Similar results were observed in other studies involving transgenic or toxin model-based systems (Bollimuntha et al., 2005; Sukumaran et al., 2018).

### TRPC3/6/7

Studies have either reported reduced or no change in the expression of TRPC3/6/7 channels in PD (Selvaraj et al., 2009, 2012; Yu et al., 2013; Streifel et al., 2014). However, the neuroprotective effects of SKF-96365, a non-selective TRPC3/6/7 blocker in the MPP^+^ model suggests a need to validate these findings (Chen et al., 2013). Therefore, challenging the reproducibility of these observations in other model systems could help resolve these concerns in the near future.

### TRPA1

Expression of TRPA channels has not been directly correlated to the pathophysiology of PD. However, it was observed that apomorphine which is an approved medication for PD is a bimodal modulator of TRPA1 channels, the only known member of the TRPA subfamily (Schulze et al., 2013). Apomorphine is often used as an add-on therapy for the management of “off” episodes in PD but exhibits adverse effects like nausea and local reactions at the injection site (Carbone et al., 2019). Schulze et al. (2013) demonstrated with the help of cultured dorsal route ganglion (DRG) neurons and in the enterochromaffin model cell line QGP-1, that these adverse effects might be attributed to the activation of TRPA1 channels by apomorphine. Therefore, strategies targeting TRPA1 channels could be useful for the management of side-effects associated with apomorphine and other currently available medications for PD.

## Challenges in the Development of TRP Channel Modulators as Drug Candidates

TRP channels are involved in the regulation of a number of physiological processes in different organs of the body. Though alteration of their functions in some of the brain areas may be involved in the pathophysiology of PD, targeting these alterations in a selective and specific manner poses a greater challenge. This was evident during the studies of TRPV1 modulators in other conditions like pain where a number of clinical trials were terminated because of the off-target effects (Krarup et al., 2011; Rowbotham et al., 2011). Encouraging results obtained in rodent studies with these agents couldn’t be replicated in patients due to a number of side effects like a diminished response to damaging heat, alteration in body temperature and reduced perception of taste were observed (Gavva et al., 2008). Therefore, management of safety and toxicity still remain the biggest hurdle for the translational success of TRP channel modulators. Another largely neglected area of research is the organellar distribution of TRP channels where they are coupled together to perform a plethora of unknown functions (Zhang et al., 2018). Besides, a report has also highlighted that the neuroprotection accorded by these agents is sex-specific which adds further complexity to the clinical development of these agents (Jia et al., 2011). Additionally, a number of agonists and antagonists of the same TRPs like TRPV1 and TRPM8 are together being investigated in the clinical trials which tell us that there is a lot more to the story than what we understand at present (Moran et al., 2011; De Caro et al., 2019; Garami et al., 2020). This further raises the possibility of these modulators to be working through other unknown mechanisms that have not been investigated till date. To develop the understanding, several transgenic mice (TRP knock in and knock out) were made, but high embryonic or postnatal lethality has largely limited their use. This has further pushed back the progress of this field and necessitated the use of alternative approaches (Park et al., 2011; Woudenberg-Vrenken et al., 2011). Another pitfall in TRP research is the lack of selective agents as activators or inhibitors for any of the ion channel. As the structure and function of TRP channels are conserved across species, preclinical and animal data using selective agents could be highly useful for the further development of these agents (Hof et al., 2019). Therefore, increased knowledge of the TRP structure is also required and needs to be studied with the help of new approaches.

Despite these limitations, there are a number of clinically approved agents like probenecid and tranilast (TRPV2), glibenclamide (TRPM4), flufenamic acid and clotrimazole (TRPM2) and menthol (TRPM8) which have also been reported to modulate TRP channel activity. There are proof of concept studies which have found them to be effective in different CNS disorders (Thapak et al., 2020a; Zubov et al., 2020). Hence, it is possible that TRP channels may be indistinctly important for brain functions, and these molecules may pave the way for a completely new class of drugs for brain-related disorders including PD.

## Conclusion

TRP channels perform a plethora of physiological functions in the CNS. The increased understanding of their role has led several researchers to investigate their likely contribution in the pathophysiology of neurodegenerative disorders, including PD. Moreover, their regulatory function in ionic homeostasis has made TRP channels an attractive pharmacological target where Ca^2+^ dyshomeostasis, oxidative stress and excitotoxicity mediated neuronal death are reported. The current review has presented a brief overview of the reports which have studied the pathophysiological involvement of TRP channels in PD. Additionally, beneficial effects of many of the pharmacological agents targeting TRP channels have also been highlighted, which suggests the possibility of a novel class of therapeutics for PD treatment in future. Amongst the agents known so far, ones targeting TRPV1, TRPM2 and TRPC1 have been most promising for PD treatment. Design of novel and selective analogs targeting these TRPs is expected to open up new avenues of research in future. Though these findings pertaining to the involvement of TRP channels in PD are encouraging, more studies highlighting the molecular mechanisms at length are required to ensure the translational success of these compounds in the clinical trials.

## Author Contributions

BV and SSS conceived the idea of the manuscript. BV wrote all the sections. SSS corrected the manuscript. Both authors approved the final proof for submission.

## Conflict of Interest

The authors declare that the research was conducted in the absence of any commercial or financial relationships that could be construed as a potential conflict of interest.

## Footnotes

1 https://www.michaeljfox.org/grant/assessing-trpml1-agonists-gba-parkinsons-disease

2 https://www.michaeljfox.org/grant/development-novel-trpml1-activator-parkinsons-disease

3 https://clinicaltrials.gov/ct2/show/NCT03321019
